# Prevalence, patterns, and associated factors of intraperitoneal adhesions among women undergoing gynaecological laparoscopy at a private endoscopic centre in Southeastern Nigeria

**DOI:** 10.3389/frph.2026.1880168

**Published:** 2026-07-24

**Authors:** Onyecherelam Monday Ogelle, Lazarus Ugochukwu Okafor, Gbenga Olorunfemi, Martin C. Andeh, John Chukwuebuka Nkesi, Chukwunonso Isaiah Enechukwu, Ifeanyi O. J. Okonkwo, Chijioke Ogomegbulam Ezeigwe, Josephat Chukwudi Akabuike, Alexander A. Mathias, Wellington-Joseph N. Ogelle, Ayotunde T. Adeyinka, Olatunde O. Alabi, Julian Mthombeni, Chidimma M. Okeke, Emmanuel C. Ogelle, Toochukwu B. Ejikeme, Christian G. Ikpeze, Chukwuemeka Okoro, Emmanuel Chukwubuikem Egwuatu, Vincent C. Ani, George Uchenna Eleje, Ikechukwu I. Mbachu, Joseph O. Ugboaja, Anthony O. Igwegbe, Nnabuike C. Ngene

**Affiliations:** 1 Department of Obstetrics and Gynaecology, Nnamdi Azikiwe University, Awka, Nigeria; 2 Department of Obstetrics and Gynaecology, Nnamdi Azikiwe University Teaching Hospital, Nnewi, Nigeria; 3 Blessed Specialist Hospital, Nkpor, Nigeria; 4 Division of Epidemiologist and Biostatistics, School of Public Health, University of Witwatersrand, Johannesburg, South Africa; 5 Department of Biomedical Science, Faculty of Health Sciences, University of Johannesburg, Johannesburg, South Africa; 6 Department of obstetrics and Gynaecology, Odumegwu Ojukwu University, Awka, Nigeria; 7 Department of Obstetrics and Gynaecology, Odumegwu Ojukwu University Teaching Hospital, Awka, Nigeria; 8 Department of Obstetrics and Gynaecology, Asaba Specialist Hospital, Asaba, Nigeria; 9 Department of Obstetrics and Gynaecology, College of Medical and Health Sciences, Novena University, Ogume, Nigeria; 10 Department of Obstetrics and Gynaecology, Federal University, Lokoja, Nigeria; 11 Department of Obstetrics and Gynaecology, University of Witwatersrand, Johannesburg, South Africa

**Keywords:** endoscopy, gynecological conditions, intraperitoneal adhesion, laparoscopy, Nigeria

## Abstract

**Background:**

Intraperitoneal adhesions are a major cause of chronic pelvic pain, infertility, tubal ectopic pregnancy, intestinal obstruction and increased surgical time globally**.**

**Objective:**

To determine the prevalence, pattern and factors associated with intraperitoneal adhesions among women undergoing laparoscopy in a private hospital in Southeastern Nigeria.

**Methods:**

We conducted a retrospective analytical cross-sectional study involving women who underwent laparoscopy at a private endoscopic centre. Sociodemographic and clinical characteristics, indications, surgical history, indications for laparoscopy, and intraoperative findings were extracted from the medical records. Peritoneal adhesion index (PAI) scores were calculated for each patient; descriptive and bivariate analyses were conducted.

**Results:**

A total of 384 women were included. The mean age was 34.89 ± 6.69 years, and the median parity was 0 (IQR: 0–1). Most of the women had endoscopy on account of infertility (*n* = 307, 79.95%). The prevalence of intraperitoneal adhesions in the cohort was 49.22% (95% CI: 44.23% to 54.23%; *n* = 189/384), while the prevalence among women with previous surgery was 72.90% (*n* = 78/107). Among women who had adhesions (*n* = 189), the median PAI score was 6 (3–12) out of a total score of 30. The prevalence of adhesions was higher among women with primary infertility compared to women with secondary infertility (*p* = 0.013). Previous abdominal surgery, type of surgery, and surgical indication were also significantly associated with adhesion formation (*p* < 0.05).

**Conclusion:**

Nearly half of the women undergoing laparoscopy had intraperitoneal adhesions. Targeted preventive strategies, including minimally invasive surgical approaches and improved reproductive health services, are essential to reduce adhesion-related morbidity. Factors predictive of intraperitoneal adhesions will assist with patient counselling, choice of therapy and optimal operative planning.

## 1 Introduction

Intraperitoneal adhesions are a major source of gynaecological morbidity, formed as a result of peritoneal injury characterised by formation of fibrous bands between the peritoneal surfaces which may also attach to the abdominal organs (1, 2). Intraperitoneal adhesions are implicated in chronic pelvic pain, secondary infertility, and increased complexity of subsequent abdominopelvic surgeries (1, 2). Adhesions also account for up to 60%–90% of cases of postoperative bowel obstruction, underscoring their clinical importance (1, 2). The reported rates of adhesion formation following open surgical procedures vary widely across populations from 34% to 93.7% (3). However, following abdominal laparoscopy, the reported rate of intraperitoneal adhesion formation was 26.6% (4).

The burden of adhesion-related disease is important in gynaecological practice, where prior pelvic surgery, pelvic inflammatory disease (PID), endometriosis, and intra-abdominal infections constitute major predisposing factors (5). In sub-Saharan Africa, these risks are further amplified by a high prevalence of PID, unsafe abortion practices, delayed access to care, and increasing caesarean section rates (6, 7). Despite these risk factors, there remains a limited availability of robust epidemiological data from the region, as most available evidence was derived from high-income settings that may not accurately reflect local disease patterns.

In the Nigerian and wider sub-Saharan African context, intraperitoneal adhesions may arise from a combination of postoperative and infection-related pathways (6–8). Previous laparotomy and myomectomy may promote adhesion formation through peritoneal trauma, tissue desiccation, bleeding, fibrin deposition, and impaired fibrinolysis. Infection-related exposures, including PID and post-abortal sepsis, may similarly trigger inflammatory exudation, tubal serosal injury, and fibrotic healing (6, 7). Although these infection-related variables were not consistently documented in the present retrospective dataset, they remain biologically plausible contributors to the burden of adhesive disease in this population (6, 8, 9).

Laparoscopy is widely regarded as the gold standard for the diagnosis of intraperitoneal adhesions, allowing direct visualization and characterization of their extent, severity, and anatomical distribution (10). With the increasing adoption of minimally invasive gynaecological surgery in Nigeria, laparoscopy provides a unique opportunity to better understand the magnitude and risk factors of intraperitoneal adhesions.

This study aims to determine the prevalence, morphological patterns, and identify the clinical predictors of intraperitoneal adhesions among women undergoing laparoscopy in a Nigerian endoscopic centre. The findings are expected to bridge existing knowledge gaps, enhance preoperative risk stratification, and inform healthcare policies in low- and middle-income settings such as Nigeria.

## 2 Methods

### 2.1 Study design and setting

This was a retrospective analytical cross-sectional study conducted among women who had laparoscopy at a private endoscopic centre in Southeastern Nigeria from January 2015 to 31st May 2025. The private facility (Blessed Specialist Hospital and Maternity) is located at Nkpor, near Onitsha city, in Anambra state, Southeastern Nigeria. The hospital offers both diagnostic and operative endoscopic procedures. The consultation and surgeries were performed by experienced consultant obstetricians and gynaecologists with extensive experience in gynaecological endoscopic surgeries. The hospital also has facilities for fertility work-up and assisted reproductive technology.

### 2.2 Study population

Clinical records of all women who underwent laparoscopy during the study period were reviewed.

### 2.3 Procedure

The hospital is a high-volume endoscopic centre conducting both diagnostic and operative endoscopic procedures. The laparoscopic procedures were performed by gynaecologists with advanced endoscopy training. Before each procedure, indications were clarified, patients were counselled, written informed consent was obtained, and preoperative work-up was conducted. The procedures were performed based on indications. Findings were succinctly documented, and postoperative care was instituted.

### 2.4 Data collection

Data were extracted from the clinical records of all women who underwent laparoscopy at the centre between 1st January 2015 and 31st May 2025 using a structured Microsoft Excel spreadsheet. The extracted variables included sociodemographic characteristics, clinical history, indications for surgery, previous surgical history, and laparoscopic findings. The presence or absence of adhesions, and their anatomical location, severity and density were also recorded.

### 2.5 Peritoneal adhesion index

The intraperitoneal adhesions were quantified using the Peritoneal Adhesion Index (PAI) as described by Coccolini et al. (11). This scoring system divides the abdomen into 10 anatomical regions, each graded on a scale of 0–3 (0 = no adhesions, 1 = filmy adhesions, 2 = moderate adhesions, 3 = dense vascular adhesions), giving a total score of 0–30.

The ten regions scored (per patient) were as follows: **R1:** Anterior Abdominal Wall (ventral surface); **R2:** Left Lateral Pelvic Side Wall; **R3:** Right Lateral Pelvic Side Wall; **R4:** Vesico-uterine space (bladder region); **R5:** Pouch of Douglas (rectouterine space); **R6:** Uterine Body and Perimetrium; **R7:** Right Peritubal region (right adnexa, including right fallopian tube and ovary); **R8:** Left Peritubal region (left adnexa); **R9:** Right Periovarian region (around the right ovary); **R10:** Left Periovarian region (around the left ovary).

Each region was graded from 0 to 3 according to adhesion severity:

**0:** No adhesions visible. **1:** Filmy or mild adhesions (avascular, translucent adhesions easily separated by blunt dissection). **2:** Moderate adhesions (partially vascularized, requiring sharp dissection, and restricting organ mobility to some extent). **3:** Dense or severe adhesions (opaque, highly vascular, inseparable without risk of injury, causing complete functional restriction).

The scores were calculated from the patients' records by two researchers.

Although there is no validated severity categories/classification based on the PAI scores, we empirically categorised PAI scores into mild (1–10), moderate (11–20), and severe (21–30) adhesions for descriptive analysis.

### 2.6 Statistics

The data were exported to Stata version 16 (Stata Corp, USA) statistical software for analysis. Categorical variables were described using frequencies and percentages while continuous variables were presented as mean and standard deviation if normally distributed and median and interquartile range if not normally distributed. The prevalence of adhesion among the cohort was calculated alongside the 95% confidence interval (CI). The prevalence of PAI classification (mild:1–10; moderate: 11–20; severe: 21–30) was also calculated. Pearson's chi-square test was used to assess the association between categorical variables and presence or absence of adhesions. Student's t-test or Mann Whitney U test was used to assess the differences in the mean or median values of a continuous variable among those women with or without adhesions. Median PAI scores were also compared across groups using Mann Whitney U test or Kruskal Wallis test. A *p*-value < 0.05 was taken as statistically significant.

### 2.7 Ethical considerations

The study was commenced after obtaining ethical approval from the ethics review board of the Nnamdi Azikiwe University Teaching Hospital, NAUTH, Nnewi (Ethics number: NAUTH/CS/66/VOL.18/VER.2/111/2025/86). Ethical principles were strictly followed. The data were collected anonymously.

## 3 Results

The records of 384 women were reviewed**.** The mean age of the women was 34.89 ± 6.69 years. The median parity was 0 (0–1) and more than half of the women had secondary education (55.37%**).** Nearly all the women were married **(**91.82%**)** and the majority lived in urban settings (75.66%) (Table 1).

**Table 1:** Comparison of sociodemographic and clinical characteristics among patients with and without intraperitoneal adhesions at laparoscopy.

Characteristics	No intraperitoneal adhesions, *n* = 195	Intraperitoneal adhesions*n* = 189	Total	*P*-value
Type of infertility				
Primary	53 (27.89)	54 (29.35)	107 (28.61)	0.013^b^^,^^*^
Secondary	113 (59.47)	87 (47.28)	200 (53.48)	
No infertility	24 (12.63)	43 (23.37)	67 (17.91)	
Age category	34.63 ± 6.84	35.14 ± 6.54	34.89 ± 6.69	0.467^c^
<35	90 (48.39)	85 (46.70)	175 (47.55)	0.815^a^
35–49	94 (50.54)	96 (52.75)	190 (51.63)	
50–59	2 (1.08)	1 (0.55)	3 (0.82)	
Educational status				
No formal	0 (0.00)	1 (0.78)	1 (0.41)	0.305^a^
Primary	6 (5.31)	2 (1.55)	8 (3.31)	
Secondary	62 (54.87)	72 (55.81)	134 (55.37)	
Tertiary	45 (39.82)	54 (41.86)	99 (40.91)	
Marital status				
Married	183 (95.31)	165 (88.24)	348 (91.82)	0.027^*^^,^^a^
Separated	1 (0.52)	2 (1.07)	3 (0.79)	
Single	7 (3.65)	19 (10.16)	26 (6.86)	
Widowed	1 (0.52)	1 (0.53)	2 (0.53)	
Place of Residence				
Rural	9 (4.71)	10 (5.35)	19 (5.03)	0.401^b^
Semi-urban	42 (21.99)	31 (16.58)	73 (19.31)	
Urban	140 (73.30)	146 (78.07)	286 (75.66)	
Occupation				
Artisan	8 (4.47)	3 (1.68)	11 (3.07)	0.600^a^
Civil Servant	43 (24.02)	44 (24.58)	87 (24.30)	
Housewife	7 (3.91)	6 (3.35)	13 (3.63)	
Public Servant	2 (1.12)	5 (2.79)	7 (1.96)	
Student	15 (8.38)	21 (11.73)	36 (10.06)	
Trader	83 (46.37)	79 (44.13)	162 (45.25)	
Others	21 (11.73)	21 (11.73)	42 (11.73)	
Parity				
(median, IQR)	0 (0–1)	0 (0–1)	0 (0–1)	0.2441^d^
0	126 (66.32)	128 (71.11)	254 (68.65)	0.598^a^
1	33 (17.37)	32 (17.78)	65 (17.57)	
2	12 (6.32)	6 (3.33)	18 (4.86)	
3	6 (3.16)	7 (3.89)	13 (3.51)	
4	10 (5.26)	5 (2.78)	15 (4.05)	
≥5	3 (1.58)	2 (1.11)	5 (1.35)	
Parity				
0	126 (66.32)	128 (71.11)	254 (68.65)	0.320^b^
≥1	64 (33.68)	52 (28.89)	116 (31.35)	
Number of previous miscarriages				
(median, IQR)	0 (0–1)	0(0–1)	0(0–1)	
0	93(51.38)	99(55.62)	192(53.48)	0.421^b^
≥1	88(48.62)	79(44.38)	167(46.52)	

a Fischer's exact test.

b Pearson's Chi-square test.

c Student's t-test.

d Mann Whitney U test.

* Statistically significant at *P*-value < 0.05.

From Table 1, history of infertility (*p* = 0.013) and marital status (*P* = 0.027) were associated with the presence of intraperitoneal adhesion. From the bivariate analysis, the prevalence of intraperitoneal adhesion was higher among women with history of primary infertility as compared to women with secondary infertility (50.48% vs. 43.5%, *P*-value = 0.013), while the prevalence of intraperitoneal adhesion was higher among single women as compared to married women (73.08% vs. 47.41%, *P*-value = 0.027). There were no statistically significant relationships between age (*p* = 0.815), educational status (*P* = 0.305), and parity (*p* = 0.320) and intraperitoneal adhesions.

**Figure 1:**
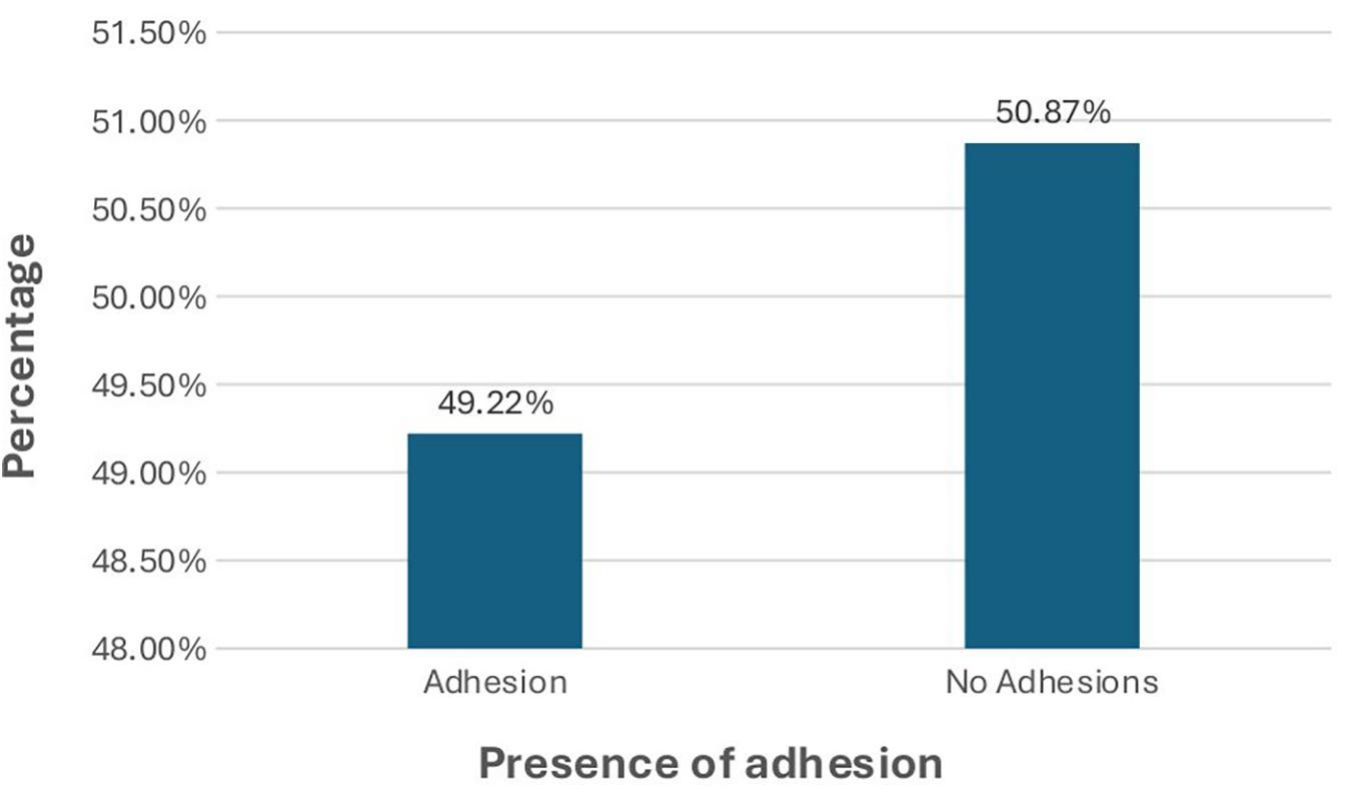
The prevalence of intraperitoneal adhesions among the cohort of women.

The prevalence of intraperitoneal adhesions among the cohort was 49.22% (95% CI: 44.23% to 54.23%; *n* = 189/384). (Figure 1)

From Table 2, there was a statistically significant relationship between previous surgery (*P* < 0.001), type of surgery (*P* < 0.001), indications for surgery (*p* = 0.009) and prevalence of intraperitoneal adhesions.

**Table 2:** Association between intraperitoneal adhesions and factors related to previous surgery.

Characteristics	No intraperitoneal adhesions, *n* = 195	Intraperitoneal adhesions *n* = 189	Total *N* = 384	*P*-value
Previous surgery				
No	152 (83.98)	100 (56.18)	252 (70.19)	<0.001*^b^
Yes	29 (16.02)	78 (43.82)	107 (29.81)	
Nature of tubal occlusion				
Bilateral occlusion	57 (29.23)	65 (34.39)	122 (31.77)	0.175^b^
Unilateral occlusion	22 (11.28)	29 (15.34)	51 (13.28)	
No occlusion	116 (59.49)	95 (50.26)	173 (45.05)	
Type of Previous surgery				
Laparoscopy	5 (17.24)	4 (5.26)	9 (8.57)	<0.001*^b^
Laparotomy	15 (51.72)	67 (88.16)	82 (78.10)	
Mini-Laparotomy	9 (31.03)	5 (6.58)	14 (13.33)	
Type of incision at previous open surgery				
Gridiron	2 (10.00)	0 (0.00)	2 (2.53)	0.116^a^
Lateral	2 (10.00)	6 (10.17)	8 (10.13)	
Paramedian	1 (5.00)	0 (0.00)	1 (1.27)	
Pfannenstiel	8 (40.00)	32 (54.24)	40 (50.63)	
Sub-umbilical midline	3 (15.00)	8 (13.56)	11 (13.92)	
Others	4 (20.00)	13 (22.03)	17 (21.52)	
Indication for previous surgery				
Appendicitis	21 (38.89)	17 (11.81)	38 (19.19)	<0.001*^a^
Ectopic pregnancy	6 (11.11)	8 (5.56)	14 (7.07)	
Endometriosis	0 (0.00)	2 (1.39)	2 (1.01)	
Fibroids	4 (7.41)	73 (50.69)	77 (38.89)	
Ovarian	23 (42.59)	42 (29.17)	65 (32.83)	
Others	0 (0.00)	2 (1.39)	2 (1.01)	

a Fischer's exact test.

b Pearson's Chi-square.

*Statistically significant at *P*-value < 0.05.

Patients with previous surgery had a higher prevalence of intraperitoneal adhesions. A previous history of laparotomy was associated with higher intraperitoneal adhesion prevalence than previous laparoscopy or mini-laparotomy. Previous surgery for fibroids was associated with the highest adhesion prevalence among recorded surgical indications.

Among women who had adhesion (*n* = 189), the median PAI score was 6 (3–12) out of a total score of 30 with a range of scores from 1 to 30. The prevalence of PAI score between 1 and 10 (mild adhesion) was 72.49%,(95%CI: 65.64% - 78.42%, *n* = 137) while the prevalence of the PAI scores between 11 and 20 (moderate adhesion) was 17.99%, (95%CI: 13.12% - 24.17%, *n* = 34) while women with prevalence of PAI scores of 21–30 (severe) was 9.52%, (95%CI: 6.06% - 14.65%, *n* = 18)

From Table 3, there was a statistically significant relationship between PAI scores and indication for previous surgery (*P*-value = 0.0132). Women who had surgery for ectopic pregnancy [2.5(1.5–6.5)] had the least median PAI scores, followed by women who had surgery for ovarian cyst [4(3–9)], appendectomy [5(4–8)], and myomectomy [10(7–18)]. The highest median PAI scores of adhesions was recorded among women who had endometriosis surgeries [15(15–15)].

**Table 3:** Association between severity scores and factors related to previous surgery.

Characteristics	Peritoneal Adhesion Index (PAI) Score, *n* = 189	
	Median, IQR	*P*-value
Type of infertility		
Primary	7 (4–12)	0.1802^k^
Secondary	5 (2–12)	
No infertility	6 (2–10)	
**Marital status**		0.1120^k^
Married	6 (3–11)	
Separated	15 (9–21)	
Single	8 (4–24)	
Widowed	2 (2–2)	
Previous pregnancy, Miscarriage, Ectopic pregnancy		
0	4 (3–8)	0.5667^m^
≥1	7 (2–16)	
Previous surgery		
No	6 (2–10.5)	0.0768^m^
Yes	7 (3–14)	
Type of Previous surgery		
Laparoscopy	5 (5–6)	0.6470^k^
Laparotomy	9.5 (6.5–17)	
Mini-Laparotomy	7 (3–15)	
Type of incision at previous open surgery		
Lateral	7.5 (5–15)	0.4181^k^
Pfannenstiel	6.5 (3–10.5)	
Sub-umbilical midline	11.5 (4–22.5)	
Others	7 (4–9)	
Gridiron	-	
Paramedian	-	
Indication for previous surgery		
Ectopic pregnancy	2.5 (1.5–6.5)	0.0132*^k^
Ovarian cyst	4 (3–9)	
Appendicitis	5 (4–8)	
Fibroids	10 (7–18)	
Endometriosis	15 (15–15)	
Others	4 (4–4)	

k, Kruskal–Wallis test; m, Mann–Whitney U-test; *Statistically significant at *p* < 0.05.

The median PAI scores were the highest among women who had sub-umbilical midline incision [11.5(4–22.5)] followed by Lateral incision [7.5(5–15)] and the Pfannenstiel incision [6.5(3–10.5)]. This relationship was not statistically significant. The median PAI score after laparoscopy was the lowest while median PAI score after laparotomy [9.5(6.5–17)] was the highest. The median PAI score was the lowest among women who had secondary infertility [5(2–12)], while median PAI score for women with primary infertility was highest [7 (4–12)] (Table 3).

From Table 4, nearly a similar proportion of the right (48.41%) and left (45.83%) fallopian tubes were abnormal. There was more prevalence of moderate to severe restricted mobility on the right tube (84.78%) as compared to the left tubes (81.43%). Furthermore, there were complete peri-tubal adhesions of the right tubes in 28% of cases while complete adhesion occurred in 30.65% of cases on the left tubes.

**Table 4:** Characterisation of fallopian tubal adhesions at laparoscopy.

Adhesion abnormalities	Right fallopian tubes		Left fallopian tubes	
	Frequency	Percentage	Frequency	Percentage
Status of tubes				
Abnormal	183	48.41	165	45.83
Normal	195	51.59	195	54.17
Restricted mobility of fallopian tube				
Mild	14	15.22	13	18.57
Moderate	40	43.48	33	47.14
Severe	38	41.30	24	34.29
Peri-tubal adhesions of each fallopian tube				
Complete	21	28.00	19	30.65
Dense	18	24.00	13	20.97
Filmy	7	9.33	2	3.23
Partial	29	38.67	28	45.16
Obscurity of fallopian tube				
Complete	38	49.35	29	55.77
Partial	39	50.65	23	44.23
Obscurity of fallopian tube if adhesion				
Complete	89	86.41	85	81.73
Partial	14	13.59	19	18.27
Site of obstruction of each fallopian tube				
Ampullary	6	7.06	2	2.90
Ampullary, and Cornual	0	0.0	1	1.45
Ampullary, and Fimbrial	1	1.18	1	1.45
Cornual	44	51.76	40	57.97
Fimbrial	16	18.82	10	14.49
Isthmic	17	20.00	15	21.74
Isthmial, and Cornual	1	1.18	0	0.0
Beaded fallopian tube				
No	128	79.01	129	83.23
Yes	34	20.99	26	16.77
Foreshortened fallopian tube				
No	133	86.93	132	89.19
Yes	20	13.07	16	10.81
Degree of distortion of fallopian tube				
Mild	8	11.11	9	14.06
Moderate	39	54.17	35	54.69
Severe	25	34.72	20	31.25
Functional status of fallopian tube				
Absent	86	35.25	78	32.77
Mild	16	6.56	10	4.20
Moderate	10	4.10	9	3.78
Normal	123	50.41	134	56.30
Severe	9	3.69	7	2.94

Table 5 below shows the degree of infiltration and organs affected by the adhesions. The highest prevalence of intraperitoneal adhesions occurred around the omentum only (48.41%). Furthermore, all the reported cases of adhesion had involvement of abdominal structures.

**Table 5:** Degree of infiltration of adhesion.

Tissue type	Frequency	Percentage
Omental	61	48.41
Appendiceal	10	7.94
Caecal	5	3.97
Peri hepatic	3	2.38
Sigmoidal	9	7.14
Caecal, appendiceal	2	1.59
Caecal, appendiceal, sigmoidal	1	0.79
Caecal, ileal, sigmoidal	4	3.17
Ileal, uterine	2	1.59
Ileal, sigmoidal	1	0.79
Omental, appendiceal	2	1.59
Omental, caecal. ovarian	1	0.79
Omental, caecal, appendiceal, ileal	7	5.55
Omental, caecal, ileal, sigmoidal, ovarian	8	6.35
Omental, ileal	3	2.38
Omental, ileal, sigmoidal	4	3.17
Omental, sigmoidal	3	2.38

## 4 Discussion

This study aimed to determine the prevalence and pattern of intraperitoneal adhesions among a cohort of women who had endoscopic gynaecological procedures at a specialist private hospital in Southeastern Nigeria. Our study will contribute to evidence on intraperitoneal adhesion among gynaecological patients undergoing laparoscopy.

### 4.1 Main findings

We found that the prevalence of intraperitoneal adhesions was 49.22%, and the prevalence of adhesions among women with previous surgery was 72.90%. The commonest sites of intraperitoneal adhesions were the anterior abdominal wall, and the greater omentum, appendix uterus and sigmoid colon. About 48.41% and 45.83% of the right and left fallopian tubes were distorted, respectively. Previous history of surgery, type of surgery and indication for the previous surgery were associated with the prevalence of intraperitoneal adhesion. The prevalence of intraperitoneal adhesions was higher among women with primary infertility as compared to women with secondary infertility (*p*-value = 0.013). However, most women with adhesions had mild adhesions as the median PAI score was low at 6 (3–12) out of a total score of 30. Nearly four out of five women (72.5%) with adhesion had mild adhesion (PAI score <10). Women who had surgery for ectopic pregnancy [2.5(1.5–6.5)] had the least median PAI scores, followed by women who had surgery for Ovarian cyst [4(3–9)], appendectomy [5(4–8)], and myomectomy [10(7–18)]. The highest median PAI scores of adhesions was recorded among women who had endometriosis surgeries [15(15–15)].

### 4.2 Interpretations

Our results reveal a high burden of disease, with nearly half (49.22%) of the cohort presenting with intraperitoneal adhesions. This prevalence is higher than the 26.6% reported in a similar Nigerian study (4) but remains consistent with global evidence that adhesions are a common sequela of abdominopelvic surgery and inflammatory processes (1, 2, 12). The previous study by Imaralu et al. was conducted at a teaching hospital in Southwestern Nigeria while our current study was conducted at a private specialist hospital in the Southeastern region of the country. The disparity in intraperitoneal adhesion prevalence as compared to the previous Nigerian study may reflect differences in patient selection, underlying morbidity, increasing surgical exposure, or improved diagnostic yield associated with laparoscopy (4).

The high prevalence observed highlights the magnitude of adhesion-related morbidity in this population. Although age, education, and residence were not significantly associated with adhesion in our study, previous studies found an association (4). Nonetheless, our study showed that marital status and history of infertility are associated with intraperitoneal adhesions among our cohort.

The higher prevalence of intraperitoneal adhesions among single women (73.08%) when compared with married women (47.41%) may reflect disparities in the prevalence of some risk factors for adhesions. Single women are at higher risk of engaging in unsafe sexual practices that may increase the prevalence of PID among them. Furthermore, single women may have a higher risk of unintended or unwanted pregnancies, thereby leading to increased risk of procuring unsafe abortion with its attendant pelvic complications (since induced abortion is legally restricted in Nigeria) (6, 8, 9). In contrast, married women generally had higher prevalence of ectopic pregnancy as compared with single women in Nigeria, which may suggest higher risk of adhesions among them (13). However, the low PAI score for ectopic pregnancy [2.5(1.5–6.5)] among our cohort of women may suggest only mild adhesion formation from the disease.

Our results showed that there was an association between previous abdominal surgery and adhesion formation (*p* < 0.001), consistent with documented evidence identifying prior surgery as the most significant risk factor (1, 4, 5). Adhesion-related complications, including readmissions and reoperations, have also been shown to be more frequent following open abdominal and gynaecological procedures (1, 5). Patients with a history of laparotomy had significantly higher adhesion prevalence compared to those who had minimally invasive procedures. This aligns with evidence demonstrating that open surgery causes greater peritoneal trauma and inflammatory response, leading to increased adhesion formation (1, 2, 12). In contrast, laparoscopic approaches are associated with reduced adhesion risk due to minimal tissue handling and decreased peritoneal injury (1, 10, 12, 14). The median PAI scores also confirmed this relationship as women who had laparotomy had the highest adhesion severity scores [9.5(6.5–17)] while women who had prior laparoscopy had the least score 5(5–6). Thus, access to endoscopic procedures in well-selected patients should be encouraged in the country.

The indication for prior surgery also influenced adhesion risk (*p* = 0.009). Procedures such as myomectomy (48%) and appendectomy (13.33%) have been strongly linked to higher adhesion prevalence and severity, particularly in women evaluated for infertility (3, 4). We found that women who had surgery for ectopic pregnancy [2.5(1.5–6.5)] had the least median PAI scores, followed by women who had surgery for ovarian cysts [4(3–9)], appendectomy [5(4–8)], and myomectomy [10(7–18)]. The highest median PAI scores of adhesions was recorded among women who had endometriosis surgeries [15(15–15)]. The mild adhesion from surgeries of ectopic pregnancy and ovarian cystectomy may suggest that the surgeries were meticulously done. Myomectomy and endometriosis have greater potential for severe intraperitoneal adhesions. Thus, gynaecologists should be very meticulous and ensure protocols aimed at preventing postoperative adhesions are followed strictly.

The prevalence of intraperitoneal adhesions was slightly higher among women who had primary infertility as compared to those with secondary infertility (50.48% vs. 43.5%, *p* = 0.013). Furthermore, the median PAI score was higher among women who had primary infertility as compared to women who had secondary infertility ([7(4–12)] vs [5(2–12)], suggesting that women with primary infertility had a more severe form of adhesion than women with secondary infertility. A common cause of infertility in sub-Saharan Africa and Nigeria is utero-tubal factors (3, 4, 15). Factors that are related to intraperitoneal adhesion and primary infertility include PID, pelvic sepsis, abdomino-pelvic surgeries (such as appendicectomy, and myomectomy), and endometriosis. In contrast, common causes of secondary infertility associated with intraperitoneal adhesions include prevalence of unsafe abortion, complications of caesarean section, ectopic pregnancy and sometimes PID.

Single women and women with primary infertility may likely engage in unsafe sexual practices leading to PID and its complications. Thus, our study showed that single women had higher prevalence of intraperitoneal adhesion as compared to married women. Furthermore, appendicectomy and myomectomy are usually performed among women with primary infertility in our environment. Likewise, adhesions following surgery for endometriosis are likely to have been conducted among women with primary infertility as endometriosis is a common cause of primary infertility. Our results further showed that the PAI score for appendicectomy, myomectomy and endometriosis were relatively high, showing that such procedures contributed immensely to the prevalence of moderate to severe adhesion among our cohort who are likely to have primary infertility. The reasons highlighted above may explain the slightly higher prevalence of adhesion among women with primary infertility as compared to women with secondary infertility.

The finding that the prevalence of adhesions was slightly higher among women with primary infertility than among those with secondary infertility should be interpreted cautiously because infertility type was assessed using bivariate analysis, and infection-related variables such as prior PID, STI history, unsafe abortion, and post-abortal sepsis were not consistently available for multivariable regression modelling. Although secondary infertility is traditionally associated with acquired pelvic infection and post-pregnancy or post-abortal complications, primary infertility may also be associated with undiagnosed or subclinical genital tract infection, endometriosis, prior abdominal surgery, or congenital and tubal factors. Therefore, the observed association should be regarded as an exploratory finding requiring confirmation in prospective studies with more detailed reproductive and infection-related data.

In sub-Saharan Africa, the burden of adhesions is exacerbated by high rates of PID, unsafe abortion practices, and increasing caesarean section rates (6–8). Despite some progress, unsafe abortion remains a significant contributor to pelvic infection and subsequent adhesion formation in the region (6). Although PID and unsafe abortion are important regional contributors to pelvic infection, these variables were not consistently captured in the retrospective records and could not be analysed directly. Additionally, rising caesarean section rates, particularly when not medically indicated, may further increase the risk of postoperative adhesions and their complications (7, 16).

The formation of postoperative adhesions is a complex inflammatory process involving peritoneal injury, fibrin deposition, and impaired fibrinolysis (2). Recent evidence highlights the role of peritoneal macrophages in modulating the inflammatory response and promoting fibrotic healing, which ultimately leads to adhesion formation (2). Intraperitoneal adhesion-related morbidity extends beyond infertility to include chronic pelvic pain, bowel obstruction, and increased risk of reoperation. Large cohort studies have demonstrated that adhesions significantly contribute to hospital readmissions and healthcare burden following abdominal surgery (1, 5).

Given the significant burden of adhesions, emphasis should be placed on prevention. Evidence-based prevention strategies include preference for laparoscopic techniques (1, 10, 12, 14), meticulous surgical techniques (17); gentle tissue handling, optimal haemostasis, use of adhesion barriers where appropriate, (10, 18, 19) as well as public health interventions such as improved infection prevention and access to safe abortion services (6).

The findings of this study have important implications for clinical practice, particularly in low-resource settings. Firstly, the high prevalence of adhesions highlights the need for improved preoperative risk stratification to identify high-risk patients and guide surgical planning, especially given the documented morbidity, readmissions, and re-operation burden associated with intraperitoneal adhesions. Secondly, the strong association with prior laparotomy justifies the necessity of prioritising minimally invasive surgical approaches whenever feasible, since open surgery is associated with greater peritoneal injury and adhesion formation, whereas laparoscopic approaches are likely to reduce adhesion risk through reduced tissue handling and peritoneal trauma (1, 10, 12). Thirdly, the possible contribution of infection-related factors in this regional context supports the need for better documentation of PID, post-abortal sepsis and related reproductive-health exposures in future prospective studies. In the current dataset, the strongest directly demonstrated risk signals were previous abdominal surgery, prior laparotomy, surgery for fibroids, and infertility. Given the substantial burden of adhesion-related morbidity, a comprehensive preventive strategy is essential (1, 5, 16). Such strategies should include the adoption of minimally invasive surgical techniques, meticulous intraoperative tissue handling, optimisation of haemostasis, and the use of evidence-based adhesion prevention agents where available. In addition, strengthening reproductive health services and improving access to early gynaecological care are critical to reducing the long-term impact of adhesions on fertility and quality of life.

### 4.3 Limitations

This study has several limitations. Firstly, its retrospective design may be subject to incomplete or missing data. Secondly, being a single-centre study, the findings may not be generalisable to other settings. Thirdly, the absence of multivariate analysis limits the ability to identify independent predictors of adhesion formation. Fourthly, key reproductive infection-related variables, including prior PID, unsafe abortion or post-abortal sepsis, were not consistently documented and could not be directly analysed. Finally, the small number of unmarried women and the absence of multivariable adjustment mean that the association between marital status and adhesions should be interpreted as exploratory.

### 4.4 Conclusion

Intraperitoneal adhesions occurred in nearly half of the women undergoing laparoscopy in this setting, majority of whom were investigated for infertility. History of previous abdominal or pelvic surgeries is associated with intraperitoneal adhesions formation, with women who had myomectomy or had surgery for endometriosis having a more severe form of adhesion formation. Efforts geared towards reducing adhesions from endometriosis and myomectomy such as meticulous tissue handling, optimum haemostasis, evidence-based adhesion-prevention measures, coupled with the use of minimally invasive surgical procedures can be considered.

## Data Availability

The raw data supporting the conclusions of this article will be made available by the authors, without undue reservation.
